# A Novel Perspective Linkage Between Kidney Function and Alzheimer’s Disease

**DOI:** 10.3389/fncel.2018.00384

**Published:** 2018-10-29

**Authors:** Yan Shi, Zhangsuo Liu, Yong Shen, Hanyu Zhu

**Affiliations:** ^1^Department of Nephrology, Chinese PLA General Hospital, Chinese PLA Institute of Nephrology, State Key Laboratory of Kidney Diseases, National Clinical Research Center for Kidney Diseases, Beijing, China; ^2^Department of Nephrology, The First Affiliated Hospital of Zhengzhou University, Research Institute of Nephrology, Zhengzhou University, Zhengzhou, China; ^3^Center for Advanced Therapeutic Strategies for Brain Disorders, The Roskamp Institute, Sarasota, FL, United States

**Keywords:** Alzheimer’s disease, kidney function, connection, CKD, cognitive impairment

## Abstract

It has long been believed that kidney function is linked to brain activity. Clinical studies demonstrate that patients with chronic kidney disease (CKD) are more prone to cognitive impairment and Alzheimer’s disease (AD), and the degree of cognitive impairment is closely related to CKD progression and renal failure. Moreover, the fact that cognitive function in CKD patients is significantly improved after successful kidney transplantation reveals a linkage between CKD and AD. However, the mechanisms behind this linkage are unclear. The physiological function of the kidney is to maintain the stability of the internal environment, including the cerebrovascular circulation, whereas abnormal kidney function often leads to ischemia and hypoxia. Many CKD patients experience chronic hypoxia, and many urinary toxins accumulate after renal function is impaired. In this mini review, we will propose a novel perspective on the association between AD and CKD and the connection between the kidney and brain.

## Introduction

**FIGURE 1:**
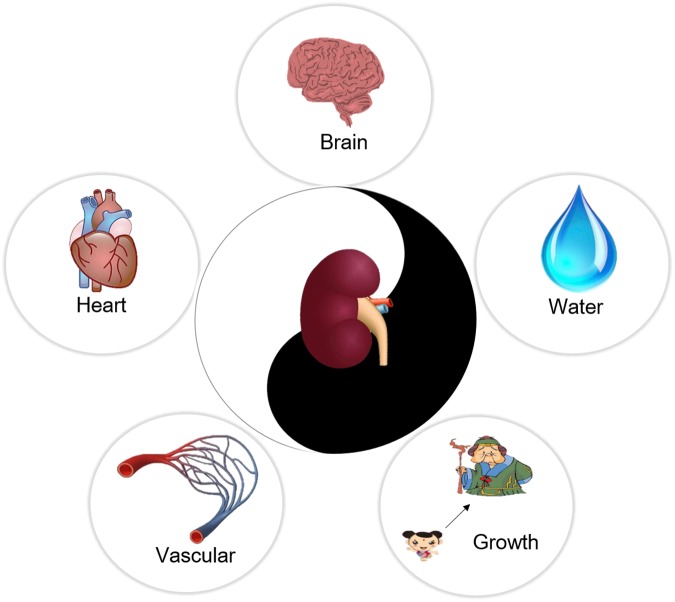
The function of kidney in Traditional Chinese Medicine. The kidney store essence, and it governs brain through the essence. Besides, the kidney governs the water, vascular, heart, and growth in ancient China.

In traditional medicine, it is believed that the kidney is the congenital foundation of the human body, and it produces and stores an “essence.” The kidney was believed to play an important role in governing water, the vascular system, the heart, growth and the brain in ancient China, as shown in Figure 1. This means the kidney modulates the brain through the “essence” it produces. The “energy” in the brain would be insufficient if the “essence” in the kidney were lacking, resulting in cognitive function decline (Li et al., 2006). Images of the brain taken by MRI of patients with kidney essence deficiency syndrome have similar imaging patterns to the brains of AD patients (Guo et al., 2017).

In 2012, a systematic meta-analysis of 54779 patients showed that the risk of cognitive impairment is significantly higher in patients with CKD than in patients without CKD (*p* < 0.001) (Etgen et al., 2012). CKD is characterized by a glomerular filtration rate (GFR) of less than 60 mL/min per 1⋅73 m^2^ or renal damage (structural or functional) for at least 3 months (Guo et al., 2017). Patients with CKD are prone to cognitive impairment at each phase (Bugnicourt et al., 2013). The lower the renal function in CKD patients is, the greater is the risk of cognitive impairment (Coppolino et al., 2018). Moreover, it has been found that a successful renal transplantation can significantly improve or even reverse cognitive impairment in patients with renal failure and the memory disorders that appeared during dialysis (Radiæ et al., 2011; Gupta et al., 2018). Furthermore, the improvement in cognitive function is closely related to renal function after transplantation and the stage of the patient’s CKD (Harciarek et al., 2009).

The kidney is the organ with the highest blood flow in the human body, as calculated by the blood flow per gram of organ. The high blood flow rate of the kidney far exceeds the needs of its own metabolism. In a sense, we understand that the kidney is an organ composed of large numbers of blood vessels that help maintain the stability of its internal environment. The blood flow through the kidneys is very fast, approximately 1200 mL per minute in a resting, healthy, normal adult, which is approximately 20–25% of the total cardiac output; moreover, the blood running through the kidneys is filtered and reabsorbed to excrete metabolic waste from the body and maintain the stability of the internal environment (the internal environment is the environment where the cells live directly in the body) (Sharma et al., 2016).

The oxygen consumption of the human brain is approximately 1/5 that of the whole body, and the amount of blood reaching the human brain is approximately 15% of the total cardiac output. The cerebral cortex is very sensitive to ischemia and hypoxia of the cerebral blood circulation (Attwell et al., 2010; Cipolla, 2016). Normal blood pressure levels allow the brain to obtain a sufficient cerebral blood supply. The balance of blood pressure relies on the complex regulatory mechanisms of neurohumoral systems, which involve all organs of the body, and the kidney is the hub of the blood pressure regulation system (Arora et al., 2015).

In the clinic, CKD is divided into 5 stages. Patients with CKD_1_ or CKD_2_ have mild renal injury, patients with CKD_3_ or CKD_4_ have moderate to severe renal injury, and stage CKD_5_ is end-stage renal disease (ESRD) or renal failure, requiring dialysis. CKD promotes the progression of AD (Etgen et al., 2012), which may be an opportunity but also a challenge for early AD diagnosis and treatment; additionally, the number of CKD patients is growing fast. For example, in 2012, the first multicenter study on CKD in China showed that the prevalence of AD was 10.8% in 50550 patients, and this rate exhibited an upward trend year by year (Zhang et al., 2012). In 2008, there were 65074 ESRD patients undergoing hemodialysis or peritoneal dialysis in China, the main factors threatening hemodialysis patients were cardiovascular and cerebrovascular diseases (i.e., stroke) (Zuo et al., 2010), and the raw annual mortality of patients undergoing maintenance hemodialysis in Beijing is gradually increasing (Cheng et al., 2012).

Alzheimer’s disease is a chronic progressive neurodegenerative disorder causing a significant cognitive deficit, and it is one of the most common types of dementia. A substantial amount of money and human resources is devoted to treating AD every year. The expenses associated with AD greatly increase the social, economic and medical burden of the disease. In 2016, there were 5.4 million AD patients who spent approximately $236 billion in the United States (Alzheimer’s Association, 2016). However, the pathogenesis of AD is still not clear, and there is no effective treatment for AD at present. However, the fact that CKD promotes the development of cognitive decline and AD results in a new field for exploring the pathogenesis of AD and sheds light on possibilities for the prevention and treatment of AD.

## Mechanisms of Cognitive Decline and AD Related to CKD

### Vascular Injury

Vascular stiffness is defined as diminished vascular elasticity, and the duration of blood vessel expansion is extended. Vascular calcification is usually characterized by the formation of vascular stiffness, manifested as the excessive deposition of calcium on the vascular walls. Vascular stiffness and calcification are common types of vascular injuries. Vascular stiffness is observed in the progression of CKD (Mukai et al., 2018), and vascular stiffness is significantly associated with cognitive impairment (Rabkin, 2018). Interestingly, proteinuria and vascular stiffness are significantly correlated in AD patients, suggesting that vascular injury is involved in some pathological processes of AD in CKD patients (Oh et al., 2016).

As mentioned above, vascular disease is significantly more common in patients with CKD, and it is mainly caused by accumulated uremic toxin due to renal dysfunction. The European Uremic Toxin Work Group has listed more than 90 kinds of uremic toxins, such as phosphorus, aluminum, and ADMA (asymmetric dimethylarginine) (Yavuz et al., 2005). The accumulation of phosphorus promotes vascular calcification (Shanahan et al., 2011). In addition, an increase in ADMA in serum is related to vascular stiffness and cerebral blood flow in healthy people; this phenomenon is generally considered a potential early biomarker and can also be explained by impairment of the endothelium dependent on vasodilatation caused by ADMA (Aldámiz-Echevarría and Andrade, 2012). Moreover, it is also important in the regulation of cerebral blood vessels (Asif et al., 2013).

The elevation of plasma Hcy (serum homocysteine) is a strong risk factor of AD and vascular disease (Li et al., 2018; Smith et al., 2018). There is a mass accumulation of Hcy in patients with CKD, probably because the Hcy clearance ability is damaged in patients with renal dysfunction (Karmin and Siow, 2018; Ohishi et al., 2018). We know that vascular injury can be caused by elevated Hcy through endothelial dysfunction in patients with CKD (Lai and Kan, 2015), suggesting that cognitive decline and/or AD is caused by Hcy through vascular endothelial dysfunction in CKD patients.

### Direct Neurotoxicity of Uremic Toxins

The massive accumulation of urinary toxins can directly act on multiple organs in the body through blood flow. For example, there is a large accumulation of parathyroid hormone (PTH) in the blood when patients have renal dysfunction. PTH can pass through the blood-brain barrier, and one study found that the PTH2 receptor was widely distributed in the central nervous system (Dobolyi et al., 2010). It has also been shown that PTH is closely related to cognitive function (Lourida et al., 2015) and that cognitive function can be improved after parathyroidectomy in patients with secondary hyperparathyroidism, suggesting that renal dysfunction-induced accumulation of PTH affects cognitive function in CKD patients.

The urinary toxin aluminum can also pass through the blood-brain barrier and act on brain tissue. Aluminum is involved in metabolic processes and redox reactions in the central nervous system, and the level of aluminum in serum is a risk factor for AD (Adlard and Bush, 2018). The accumulation of aluminum after renal dysfunction can contribute to cognitive decline and AD in CKD patients.

Cognitive function abnormalities are characteristic of hemodialysis patients, and hemodialysis patients have more cerebral atrophy, partly caused by cerebral ischemia during dialysis (McIntyre and Goldsmith, 2015; Tsuruya and Yoshida, 2018). Patients with impaired renal function are more prone to cognitive impairment, which is mainly due to renal anemia (Kurella Tamura et al., 2011) and ischemic hypoxia of cells in the brain involved in the process of cognitive impairment in CKD patients.

**FIGURE 2:**
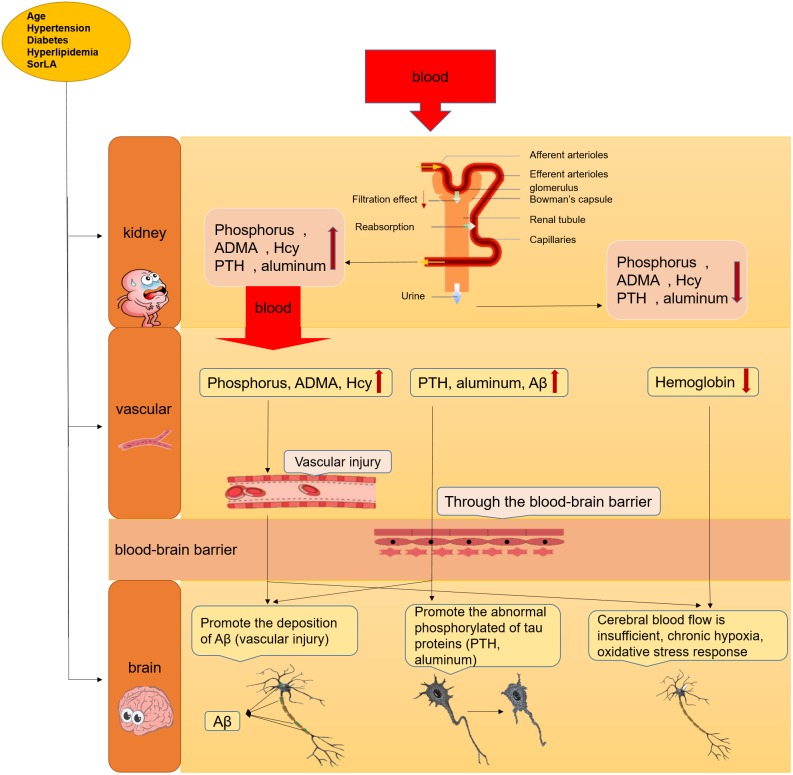
The mechanism about the cognitive decline or AD in CKD patients. The cognitive decline or AD can leaded by the accumulation of uremic toxins in serum through vascular injury and direct neurotoxicity in brain after renal failure, sunh as PTH, phosphorus, ADMA, etc.

The risks of cognitive impairment in AD patients with CKD are significantly higher than those in patients without CKD (Ito et al., 2018), not only in older CKD patients but also in young CKD patients (20–59 years old) (Hailpern et al., 2007). These risks can be explained by two factors: vascular injury and the direct neurotoxicity of uremic toxins caused by CKD. Figure 2 shows the proposed mechanism for cognitive decline in AD patients with CKD.

### Common Risk Factors

There are many common characteristics between the pathogenesis of AD and that of CKD, including vascular dysfunction or degeneration (Li et al., 2011; Xue et al., 2014), aging (Baumgart et al., 2015; Kopp, 2018), hypertension (Forbes and Cooper, 2013), diabetes (Umegaki et al., 2013) and hyperlipidemia (Reitz et al., 2011; Wan et al., 2013; Ma et al., 2016). In addition, the expression level of APP in patients with kidney disease is higher, and a key protein, SorLA (sorting protein-related receptor), that regulates APP processing is simultaneously expressed in both kidney cells and neurons, and the gene’s polymorphism is related to late-onset AD (Yarbrough, 2010).

### The Risk Assessment of AD in CKD Patients

At present, diagnosis of AD is mainly determined by physicians’ subjective experience and neuropsychological tests (Robillard et al., 2018). Unfortunately, patients are already in late stages of the disease once AD clinical diagnoses are made. However, the best time to stop the progression of AD is before the onset of clinical symptoms. It is encouraging that we now understand not only that AD is related to CKD but also that both share similar pathological processes. This relationship may provide new insight into the pathogenesis of the disease since there are a few potential markers for the diagnosis of CKD and AD. For instance, we could validate whether there are any links between the biomarkers of CKD and AD and whether the biomarkers for CKD can be used for the diagnosis of AD.

## The Potential Markers Related to AD

### Aβ Protein, Aβ40/ Aβ42

The traditional pathological hallmarks in the AD brain are neuron/synapse loss and the presence of large numbers of senile plaques and neurofibrillary tangles in the cerebral cortex and hippocampus. Aβ protein is a major component of senile plaques and can be secreted directly into cerebrospinal fluid (CSF) by neurons (Blennow et al., 2010).

Interestingly, it was also found that serum Aβ levels were significantly higher in CKD patients (Kanemaru et al., 2016), possibly due to the decreased clearance of Aβ protein in the blood of CKD patients, suggesting that cognitive decline and AD related to CKD can be affected by Aβ protein in CKD patients. Many studies have reported that Aβ40/Aβ42 levels were significantly increased in the CSF of AD patients, and this parameter is very sensitive and specific (Hansson et al., 2010; Blennow and Zetterberg, 2018).

### Tau Protein

Neurofibrillary tangles are another characteristic pathological change in the AD brain, and abnormally phosphorylated tau proteins are the main constituent proteins of neurofibrillary tangles (Brici et al., 2018). An increase in tau protein/phosphorylated tau (p-tau) in the CSF is a sign of AD (Zhou et al., 2018). Moreover, studies show that changes in the tau protein can be associated with the pathology of AD after the elevation of aluminum in renal dialysis patients (CKD_5_) (Harrington et al., 1994).

### Serum Homocysteine

Serum homocysteine (Hcy) is a sulfur-containing amino acid (Brustolin et al., 2010). As mentioned above, serum Hcy clearance is significantly decreased in patients with CKD and patients already prone to high Hcy in the blood (Karmin and Siow, 2018; Tabibzadeh et al., 2018). Interestingly, serum Hcy levels were found to be significantly higher in AD patients (Baumgart et al., 2015), and serum Hcy levels are a strong and independent risk factor of AD and dementia (Xue et al., 2014). Serum Hcy is involved in the pathogenesis of AD through endothelial cell dysfunction and small blood vessels by oxidative stress (Baumgart et al., 2015). Moreover, CKD is an independent risk factor for increased serum Hcy levels (Choi et al., 2014), and the elevation of Hcy caused by CKD was found to be associated with the reduction in Aβ42 in the CSF (Hansson et al., 2010). Thus, serum Hcy levels may be a novel maker of AD in patients with CKD.

## The Potential Biomarkers Related to CKD

### Proteinuria, Creatinine, eGFR

Proteinuria and eGFR are common indicators for renal function (Jha et al., 2013; Conkar et al., 2018). Proteinuria is often found in patients with CKD. Under pathological conditions, the amount of protein in the urine increases, mainly due to damaged glomerular filtration barriers (endothelial cells, podocytes, glomerular basement membrane) and their increased permeability.

Glomerular filtration function is expressed by the GFR, [mL/(min 1.73 m^2^)], and the GFR is the amount of fluid produced by the two kidneys per unit of time. The GFR is currently calculated by the endogenous marker creatinine in the clinic. Creatinine is excreted by renal tubules and is not metabolized by the kidneys. In patients with CKD, excretion of creatinine by renal tubules is lower, the GFR is lower, and the serum concentration of creatinine is higher.

Proteinuria and eGFR are closely related to cognitive decline (Kurella Tamura et al., 2011; Etgen et al., 2012; Ito et al., 2018). One study found that the increase in ptau in the CSF and Hcy in the serum was significantly associated with a decrease in GFR in AD patients (Kanemaru et al., 2016), suggesting that there is a connection between CKD and the increase in ptau in the CSF. Additionally, the creatinine level was closely related to cognitive impairment (McAdams-DeMarco et al., 2016).

### Hemoglobin Concentration

Erythropoietin (EPO) is mainly produced in the kidney (Bachmann et al., 1993); EPO promotes the formation of red blood cells and maintains normal hemoglobin concentrations. The concentrations of hemoglobin and EPO in the serum of CKD patients are significantly lower, and CKD patients are more prone to anemia and anemic hypoxia (Kutuby et al., 2015).

A large cohort study with 3591 subjects found that CKD patients were more likely to exhibit cognitive decline. However, after adjusting for hemoglobin concentration, this link became weak or disappeared (Kurella Tamura et al., 2011), suggesting that this strong correlation was mainly caused by the change in hemoglobin concentration in CKD patients. The level of hemoglobin in the serum was significantly correlated with cognitive impairment. Additionally, studies have demonstrated that EPO can have neuroprotective effects (Fan et al., 2012), the production of EPO can be stimulated by anemic hypoxia in the brain, and anemia is an independent risk factor of cognitive decline (Sousa et al., 2018).

### Cystatin C

The concentration of cystatin C is mainly determined by the GFR because the kidney is the only organ that cleanses cystatin C (Krawczeski et al., 2010). Research confirms that the gene encoding cystatin C (CST3) is a susceptible gene of late-onset AD. In addition, it has been found that cystatin C and Aβ colocalize in the amyloid deposition of brain parenchyma and cerebral vessels of AD patients (Sastre et al., 2004), and this study also found colocalization of cystatin C and APP in transfected cells and cell membranes through immunofluorescence analysis and a combination of cystatin C, full-length APP and secreted βAPP through western blots of immunoprecipitated cell lysates and culture proteins (Finckh et al., 2000).

The levels of cystatin C in the plasma of AD patients were lower than those in the age-matched control group (Chuo et al., 2007). It is unclear whether the deposition of cystatin C in the brain leads to a decrease in cystatin C in the peripheral plasma. Furthermore, if cystatin C concentrations increase in the plasma of CKD patients, it is unclear whether this would help promote binding and precipitation between cystatin C and APP in the brain. The level of cystatin C in the serum may be a potential new biomarker of AD and cognitive decline in CKD patients.

### Urotoxin

Uremic toxins have been among the most studied factors leading to uremia since 1840 when P.A. Piorry and D.I. Heritier proposed the concept of uremia (Richet, 1998). It has been found that there are more than 90 kinds of urinary toxins in uremic patients (Duranton et al., 2012), but their impact on the body is not clear. In recent years, increasing attention has been focused on small molecule toxins and intermediate-sized molecule toxins, such as PTH (Movilli et al., 2011; Neirynck et al., 2013), phosphorus, aluminum and ADMA. Some studies have found a significant increase in PTH in the blood of patients with CKD (Souberbielle et al., 2010; Lishmanov et al., 2012), and PTH is closely related to a decline in cognitive function (Lourida et al., 2015). Additionally, phosphorus and ADMA have confirmed involvement in the progress of vascular injury (van Kuijk et al., 2010; Shanahan et al., 2011). Some scholars have found that the level of aluminum is a risk factor for AD (Walton, 2013).

summary, numerous epidemiological studies demonstrate that there is a high incidence of cognitive impairment or AD-like dementia in CKD patients of all ages. This discovery highlights a new direction for the diagnosis and potential treatment of both CKD and AD. Vascular injury and the direct neurotoxicity of uremic toxins caused by renal dysfunction are the most reasonable mechanisms of the effects of CKD in AD patients. We might obtain unexpected results if we focus our attention on changes occurring in both the brain and the kidneys in further studies in the prevention and treatment of CKD and AD.

## Author Contributions

YaS and ZL contributed to drafting and revising this manuscript. YoS and HZ designed and revised this manuscript.

## Conflict of Interest Statement

The authors declare that the research was conducted in the absence of any commercial or financial relationships that could be construed as a potential conflict of interest.

## References

[B1] Adlard P. A. Bush A. I. (2018). Metals and Alzheimer’s disease: how far have we come in the clinic? *J. Alzheimers Dis.* 62 1369–1379. 10.3233/JAD-170662 29562528 PMC5870044

[B2] Aldámiz-Echevarría L. Andrade F. (2012). Asymmetric dimethylarginine, endothelial dysfunction and renal disease. *Int. J. Mol. Sci.* 13 11288–11311. 10.3390/ijms130911288 23109853 PMC3472745

[B3] Alzheimer’s Association (2016). 2016 Alzheimer’s disease facts and figures. *Alzheimers Dement.* 12 459–509. 10.1016/j.jalz.2016.03.001 27570871

[B4] Arora P. Golzy M. Patel N. Quigg R. Carter R. L. Lohr J. W. (2015). Renin-angiotensin-aldosterone system blockers in elderly adults with chronic kidney disease without diabetes mellitus or proteinuria. *J. Am. Geriatr. Soc.* 63 2478–2484. 10.1111/jgs.13842 26691698

[B5] Asif M. Louis S. R. McEvoy M. Mangoni A. (2013). Asymmetric dimethylarginine: a possible link between vascular disease and dementia. *Curr. Alzheimer Res.* 10 347–356. 10.2174/1567205011310040001 23036019

[B6] Attwell D. Buchan A. M. Charpak S. Lauritzen M. Macvicar B. A. Newman E. A. (2010). Glial and neuronal control of brain blood flow. *Nature* 468 232–243. 10.1038/nature09613 21068832 PMC3206737

[B7] Bachmann S. Le Hir M. Eckardt K. U. (1993). Co-localization of erythropoietin mRNA and ecto-5’-nucleotidase immunoreactivity in peritubular cells of rat renal cortex indicates that fibroblasts produce erythropoietin. *J. Histochem. Cytochem.* 41 335–341. 10.1177/41.3.8429197 8429197

[B8] Baumgart M. Snyder H. M. Carrillo M. C. Fazio S. Kim H. Johns H. (2015). Summary of the evidence on modifiable risk factors for cognitive decline and dementia: a population-based perspective. *Alzheimers Dement.* 11 718–726. 10.1016/j.jalz.2015.05.016 26045020

[B9] Blennow K. Hampel H. Weiner M. Zetterberg H. (2010). Cerebrospinal fluid and plasma biomarkers in Alzheimer disease. *Nat. Rev. Neurol.* 6 131–144. 10.1038/nrneurol.2010.4 20157306

[B10] Blennow K. Zetterberg H. (2018). Biomarkers for Alzheimer disease – current status and prospects for the future. *J. Int. Med.* 10.1111/joim.12816[Epub ahead of print]. 30051512

[B11] Brici D. Götz J. Nisbet R. M. (2018). A novel antibody targeting tau phosphorylated at serine 235 detects neurofibrillary tangles. *J. Alzheimers Dis.* 61 899–905. 10.3233/JAD-170610 29332046

[B12] Brustolin S. Giugliani R. Félix T. M. (2010). Genetics of homocysteine metabolism and associated disorders. *Braz. J. Med. Biol. Res.* 43 1–7. 10.1590/S0100-879X2009007500021 19967264 PMC3078648

[B13] Bugnicourt J. M. Godefroy O. Chillon J. M. Choukroun G. Massy Z. A. (2013). Cognitive disorders and dementia in CKD: the neglected kidney-brain axis. *J. Am. Soc. Nephrol.* 24 353–363. 10.1681/ASN.2012050536 23291474

[B14] Cheng X. Nayyar S. Wang M. Li X. Sun Y. Huang W. (2012). Mortality rates among prevalent hemodialysis patients in Beijing: a comparison with USRDS data. *Nephrol. Dial. Transplant.* 28 724–732. 10.1093/ndt/gfs326 22907953

[B15] Choi S. T. Kim J. S. Song J. S. (2014). Elevated serum homocysteine levels were not correlated with serum uric acid levels, but with decreased renal function in gouty patients. *J. Korean Med. Sci.* 29 788–792. 10.3346/jkms.2014.29.6.788 24932079 PMC4055811

[B16] Chuo L. J. Sheu W. H. Pai M. C. Kuo Y. M. (2007). Genotype and plasma concentration of cystatin C in patients with late-onset Alzheimer disease. *Dement. Geriatr. Cogn. Disord.* 23 251–257. 10.1159/000100021 17310123

[B17] Cipolla M. J. (2016). The cerebral circulation: colloquium series on integrated systems physiology: from molecule to function to disease. *Morgan Claypool Life Sci.* 8 1–80.

[B18] Conkar S. Mir S. Karaslan F. N. Hakverdi G. (2018). Comparing different estimated glomerular filtration rate equations in assessing glomerular function in children based on creatinine and cystatin C. *J. Clin. Lab. Anal.* 10.1002/jcla.22413 [Epub ahead of print]. 29484708 PMC6817194

[B19] Coppolino G. Bolignano D. Gareri P. Ruberto C. Andreucci M. Ruotolo G. (2018). Kidney function and cognitive decline in frail elderly: two faces of the same coin? *Int. Urol. Nephrol.* 50 1505–1510. 10.1007/s11255-018-1900-3 29868939

[B20] Dobolyi A. Palkovits M. Usdin T. B. (2010). The TIP39–PTH2 receptor system: unique peptidergic cell groups in the brainstem and their interactions with central regulatory mechanisms. *Prog. Neurobiol.* 90 29–59. 10.1016/j.pneurobio.2009.10.017 19857544 PMC2815138

[B21] Duranton F. Cohen G. De Smet R. Rodriguez M. Jankowski J. Vanholder R. (2012). Normal and pathologic concentrations of uremic toxins. *J. Am. Soc. Nephrol.* 23 1258–1270. 10.1681/ASN.2011121175 22626821 PMC3380651

[B22] Etgen T. Chonchol M. Förstl H. Sander D. (2012). Chronic kidney disease and cognitive impairment: a systematic review and meta-analysis. *Am. J. Nephrol.* 35 474–482. 10.1159/000338135 22555151

[B23] Fan X. van Bel F. van der Kooij M. A. (2012). Hypothermia and erythropoietin for neuroprotection after neonatal brain damage. *Pediatr. Res.* 73 18–23. 10.1038/pr.2012.139 23085819

[B24] Finckh U. von der Kammer H. Velden J. Michel T. Andresen B. Deng A. (2000). Genetic association of a cystatin C gene polymorphism with late-onset Alzheimer disease. *Arch. Neurol.* 57 1579–1583. 10.1001/archneur.57.11.1579 11074789

[B25] Forbes J. M. Cooper M. E. (2013). Mechanisms of diabetic complications. *Physiol. Rev.* 93 137–188. 10.1152/physrev.00045.2011 23303908

[B26] Guo Z. Liu X. Cao Y. Hou H. Chen X. Chen Y. (2017). Common 1 H-MRS characteristics in patients with Alzheimer’s disease and vascular dementia diagnosed with kidney essence deficiency syndrome: a preliminary study. *Altern. Ther. Health Med.* 23 12–18. 28236618

[B27] Gupta A. Thomas T. S. Klein J. A. Montgomery R. N. Mahnken J. D. Johnson D. K. (2018). Discrepancies between perceived and measured cognition in kidney transplant recipients: implications for clinical management. *Nephron* 138 22–28. 10.1159/000481182 29049997 PMC5828957

[B28] Hailpern S. M. Melamed M. L. Cohen H. W. Hostetter T. H. (2007). TH Moderate chronic kidney disease and cognitive function in adults 20 to 59 years of age: third national health and nutrition examination survey (NHANES III). *J. Am. Soc. Nephrol.* 18 2205–2213. 10.1681/ASN.2006101165 17554148

[B29] Hansson O. Zetterberg H. Vanmechelen E. Vanderstichele H. Andreasson U. Londos E. (2010). Evaluation of plasma Aβ 40 and Aβ 42 as predictors of conversion to Alzheimer’s disease in patients with mild cognitive impairment. *Neurobiol. Aging* 31 357–367. 10.1016/j.neurobiolaging.2008.03.027 18486992

[B30] Harciarek M. Biedunkiewicz B. Lichodziejewska-Niemierkom M. Debska-Slizień A. Rutkowski B. (2009). Cognitive performance before and after kidney transplantation: a prospective controlled study of adequately dialyzed patients with end-stage renal disease. *J. Int. Neuropsychol. Soc.* 15 684–694. 10.1017/S1355617709990221 19570307

[B31] Harrington C. R. Wischik C. M. McArthur F. K. Taylor G. A. Edwardson J. A. Candy J. M. (1994). Alzheimer’s-disease-like changes in tau protein processing: association with aluminium accumulation in brains of renal dialysis patients. *Lancet* 343 993–997. 10.1016/S0140-6736(94)90124-4 7909090

[B32] Ito H. Antoku S. Mori T. Nakagawa Y. Mizoguchi K. Matsumoto S. (2018). Association between chronic kidney disease and the cognitive function in subjects without overt dementia. *Clin. Nephrol.* 89 330–335. 10.5414/CN109188 29057735

[B33] Jha V. Garcia-Garcia G. Iseki K. Li Z. Naicker S. Plattner B. (2013). Chronic kidney disease: global dimension and perspectives. *Lancet* 382 260–272. 10.1016/S0140-6736(13)60687-X 23727169

[B34] Kanemaru K. Kanemaru A. Murayama S. (2016). Association between renal functions and csf biomarkers in alzheimer’s disease. *Alzheimer’s Dement. J. Alzheimer’s Assoc.* 12:665 10.1016/j.jalz.2016.06.1508

[B35] Karmin O. Siow Y. L. (2018). Metabolic imbalance of homocysteine and hydrogen sulfide in kidney disease. *Curr. Med. Chem.* 25 367–377. 10.2174/0929867324666170509145240 28486919

[B36] Kopp J. B. (2018). Global glomerulosclerosis in primary nephrotic syndrome: including age as a variable to predict renal outcomes. *Kidney Int.* 93 1043–1044. 10.1016/j.kint.2018.01.020 29680021

[B37] Krawczeski C. D. Vandevoorde R. G. Kathman T. Bennett M. R. Woo J. G. Wang Y. (2010). Serum cystatin C is an early predictive biomarker of acute kidney injury after pediatric cardiopulmonary bypass. *Clin. J. Am. Soc. Nephrol.* 5 1552–1557. 10.2215/CJN.02040310 20538834 PMC2974393

[B38] Kurella Tamura M. Xie D. Yaffe K. Cohen D. L. Teal V. Kasner S. E. (2011). Vascular risk factors and cognitive impairment in chronic kidney disease: the Chronic Renal Insufficiency Cohort (CRIC) study. *Clin. J. Am. Soc. Nephrol.* 6 248–256. 10.2215/CJN.02660310 20930087 PMC3052213

[B39] Kutuby F. Wang S. Desai C. Lerma E. V. (2015). Anemia of chronic kidney disease. *Dis. Mon.* 61 421–424. 10.1016/j.disamonth.2015.08.002 26364946

[B40] Lai W. K. C. Kan M. Y. (2015). Homocysteine-induced endothelial dysfunction. *Ann. Nutr. Metab.* 67 1–12. 10.1159/000443041 26201664

[B41] Li J. Wang Y. J. Zhang M. Xu Z. Q. Gao C. Y. Fang C. Q. (2011). Vascular risk factors promote conversion from mild cognitive impairment to Alzheimer disease. *Neurology* 76 1485–1491. 10.1212/WNL.0b013e318217e7a4 21490316

[B42] Li L. Wei H. F. Zhang L. Chu J. Zhao L. (2006). Modern biological basis of chinese medical theory that “kidney nourishes marrow and brain is sea of marrow”. *Zhongguo Zhong Yao Za Zhi* 31 1397–1400. 17087074

[B43] Li T. Yu B. Liu Z. Li J. Ma M. Wang Y. (2018). Homocysteine directly interacts and activates the angiotensin II type I receptor to aggravate vascular injury. *Nat. Commun.* 9:11. 10.1038/s41467-017-02401-7 29296021 PMC5750214

[B44] Lishmanov A. Dorairajan S. Pak Y. Chaudhary K. Chockalingam A. (2012). Elevated serum parathyroid hormone is a cardiovascular risk factor in moderate chronic kidney disease. *Int. Urol. Nephrol.* 44 541–547. 10.1007/s11255-010-9897-2 21327525

[B45] Lourida I. Thompson-Coon J. Dickens C. M. Soni M. Kuźma E. Kos K. (2015). Parathyroid hormone, cognitive function and dementia: a systematic review. *PLoS One* 10:e0127574. 10.1371/journal.pone.0127574 26010883 PMC4444118

[B46] Ma C. Yin Z. Zhu P. Luo J. Shi X. Gao X. (2016). Blood cholesterol in late-life and cognitive decline: a longitudinal study of the chinese elderly. *Mol. Neurodegener.* 12:24. 10.1186/s13024-017-0167-y 28270179 PMC5341475

[B47] McAdams-DeMarco M. A. Bae S. Chu N. Gross A. L. Brown C. H. I. V. Oh E. (2016). Dementia and Alzheimer’s disease among older kidney transplant recipients. *J. Am. Soc. Nephrol.* 28 1575–1583. 10.1681/ASN.2016080816 27979990 PMC5407731

[B48] McIntyre C. W. Goldsmith D. J. (2015). Ischemic brain injury in hemodialysis patients: which is more dangerous, hypertension or intradialytic hypotension? *Kidney Int.* 87 1109–1115. 10.1038/ki.2015.62 25853331

[B49] Movilli E. Camerini C. Gaggia P. Poiatti P. Pola A. Viola B. F. (2011). Effect of post-dilutional on-line haemodiafiltration on serum calcium, phosphate and parathyroid hormone concentrations in uraemic patients. *Nephrol. Dial. Transplant.* 26 4032–4037. 10.1093/ndt/gfr179 21555393

[B50] Mukai H. Svedberg O. Lindholm B. Dai L. Heimbürger O. Barany P. (2018). Skin autofluorescence, arterial stiffness and Framingham risk score as predictors of clinical outcome in chronic kidney disease patients: a cohort study. *Nephrol. Dial Transplant.* 10.1093/ndt/gfx371 [Epub ahead of print]. 29378035

[B51] Neirynck N. Vanholder R. Schepers E. Eloot S. Pletinck A. (2013). An update on uremic toxins. *Int. Urol. Nephrol.* 45 139–150. 10.1007/s11255-012-0258-1 22893494

[B52] Oh Y. S. Kim J. S. Park J. W. An J. Y. Park S. K. Shim Y. S. (2016). Arterial stiffness and impaired renal function in patients with Alzheimer’s disease. *Neurol. Sci.* 37 451–457. 10.1007/s10072-015-2434-4 26684808

[B53] Ohishi T. Fujita T. Suzuki D. Nishida T. Asukai M. Matsuyama Y. (2018). Serum homocysteine levels are affected by renal function during a 3-year period of minodronate therapy in female osteoporotic patients. *J. Bone Miner Metab.* 10.1007/s00774-018-0920-5 [Epub ahead of print]. 29603071

[B54] Rabkin S. W. (2018). Is it time to utilize measurement of arterial stiffness to identify and reduce the risk of cognitive impairment? *J. Clin. Hypertens. (Greenwich)* 20 31–32. 10.1111/jch.13126 29338110 PMC8031005

[B55] Radiæ J. Ljutiæ D. Radiæ M. Kovaèiæ V. Dodig-Æurkoviæ K. Šain M. (2011). Kidney transplantation improves cognitive and psychomotor functions in adult hemodialysis patients. *Am. J. Nephrol.* 34 399–406. 10.1159/000330849 21934300

[B56] Reitz C. Brayne C. Mayeux R. (2011). Epidemiology of Alzheimer disease. *Nat. Rev. Neurol.* 7 137–152. 10.1038/nrneurol.2011.2 21304480 PMC3339565

[B57] Richet G. (1998). Early history of uremia. *Kidney Int.* 33 1013–1015. 10.1038/ki.1988.102 3292814

[B58] Robillard J. M. Lai J. A. Wu J. M. Feng T. L. Hayden S. (2018). Patient perspectives of the experience of a computerized cognitive assessment in a clinical setting. *Alzheimer’s Dementia* 4 297–303. 10.1016/j.trci.2018.06.003 30090850 PMC6077833

[B59] Sastre M. Calero M. Pawlik M. Mathews P. M. Kumar A. Danilov V. (2004). Binding of cystatin C to Alzheimer’s amyloid β inhibits in vitro amyloid fibril formation. *Neurobiol. Aging* 25 1033–1043. 10.1016/j.neurobiolaging.2003.11.006 15212828

[B60] Shanahan C. M. Crouthamel M. H. Kapustin A. Giachelli C. M. (2011). Arterial calcification in chronic kidney disease: key roles for calcium and phosphate. *Circ. Res.* 109 697–711. 10.1161/CIRCRESAHA.110.234914 21885837 PMC3249146

[B61] Sharma S. Ruebner R. L. Furth S. L. Dodds K. M. Rychik J. Goldberg D. J. (2016). Assessment of kidney function in survivors following Fontan palliation. *Congenit Heart Dis.* 11 630–636. 10.1111/chd.12358 27106111

[B62] Smith A. D. Refsum H. Bottiglieri T. Fenech M. Hooshmand B. McCaddon A. (2018). Homocysteine and dementia: an international consensus statement. *J. Alzheimers Dis.* 62 561–570. 10.3233/JAD-171042 29480200 PMC5836397

[B63] Souberbielle J. C. Roth H. Fouque D. P. (2010). Parathyroid hormone measurement in CKD. *Kidney Int.* 77 93–100. 10.1038/ki.2009.374 19812537

[B64] Sousa N. D. S. Menezes T. N. Silva N. A. Mdc E. L. Paiva A. A. (2018). [prevalence of anemia and correlation between the concentration of hemoglobin and cognitive factors among the elderly]. *Cien Saude Colet* 23 935–944. 10.1590/1413-81232018233.09082016 29538573

[B65] Tabibzadeh N. Mentaverri R. Daroux M. Mesbah R. Delpierre A. Paul J. G. (2018). Differential determinants of tubular phosphate reabsorption: insights on renal excretion of phosphates in kidney disease. *Am. J. Nephrol.* 47 300–303. 10.1159/000488864 29779025

[B66] Tsuruya K. Yoshida H. (2018). Brain atrophy and cognitive impairment in chronic kidney disease. *Contrib. Nephrol.* 196 27–36. 10.1159/000485694 30041201

[B67] Umegaki H. Hayashi T. Nomura H. Yanagawa M. Nonogaki Z. Nakshima H. (2013). Cognitive dysfunction: an emerging concept of a new diabetic complication in the elderly. *Geriatr. Gerontol. Int.* 13 28–34. 10.1111/j.1447-0594.2012.00922.x 22882533

[B68] van Kuijk J. P. Flu W. J. Chonchol M. Valentijn T. M. Verhagen H. J. Bax J. J. (2010). Elevated preoperative phosphorus levels are an independent risk factor for cardiovascular mortality. *Am. J. Nephrol.* 32 163–168. 10.1159/000315856 20606420

[B69] Walton J. R. (2013). Aluminum involvement in the progression of Alzheimer’s disease. *J. Alzheimers. Dis.* 35 7–43. 10.3233/JAD-121909 23380995

[B70] Wan J. Wang S. Haynes K. Denburg M. R. Shin D. B. Gelfand J. M. (2013). Risk of moderate to advanced kidney disease in patients with psoriasis: population based cohort study. *BMJ* 347:f5961. 10.1136/bmj.f5961 24129480 PMC3805477

[B71] Xue L. Lou Y. Feng X. Wang C. Ran Z. Zhang X. (2014). Prevalence of chronic kidney disease and associated factors among the Chinese population in Taian, China. *BMC Nephrol.* 15:205. 10.1186/1471-2369-15-205 25528680 PMC4382930

[B72] Yarbrough C. (2010). *Alzheimer’s and Kidney Disease: Common Molecular Culprit? Kidney News.* 2:9.

[B73] Yavuz A. Tetta C. Ersoy F. F. D’intini V. Ratanarat R. De Cal M. (2005). Reviews: uremic toxins: a new focus on an old subject. *Semin. Dial.* 18 203–211. 10.1111/j.1525-139X.2005.18313.x 15934967

[B74] Zhang L. Wang F. Wang L. Wang W. Liu B. Liu J. (2012). Prevalence of chronic kidney disease in China: a cross-sectional survey. *Lancet* 379 815–822. 10.1016/S0140-6736(12)60033-6 22386035

[B75] Zhou Y. Shi J. Chu D. Hu W. Guan Z. Gong C. X. (2018). Relevance of phosphorylation and truncation of tau to the etiopathogenesis of alzheimer’s disease. *Front. Aging Neurosci.* 10:27 10.3389/fnagi.2018.00027 PMC5810298 29472853

[B76] Zuo L. Wang M. Chinese Association of Blood Purification Management of Chinese Hospital Association (2010). Current burden and probable increasing incidence of ESRD in China. *Clin. Nephrol.* 74 S20–S22. 20979958

